# Cost-effectiveness of magnetic resonance-guided focused ultrasound surgery for treatment of uterine fibroids

**DOI:** 10.1111/j.1471-0528.2007.01657.x

**Published:** 2008-04-01

**Authors:** H Zowall, JA Cairns, C Brewer, DL Lamping, WMW Gedroyc, L Regan

**Affiliations:** a McGill University Montreal, Quebec, Canada; b Health Services Research Unit, London School of Hygiene & Tropical Medicine London, UK; c Zowall Consulting Montreal, Quebec, Canada; d Departments of Magnetic Resonance Imaging, St Mary's Hospital and Imperial College School of Medicine London, UK; e Departments of Academic Obstetrics and Gynaecology, St Mary's Hospital and Imperial College School of Medicine London, UK

**Keywords:** Cost-utility analysis, focused ultrasound surgery, uterine fibroids

## Abstract

**Objective:**

To estimate the cost-effectiveness of a treatment strategy for symptomatic uterine fibroids, which starts with Magnetic Resonance-guided Focused Ultrasound Surgery (MRgFUS) as compared with current practice comprising uterine artery embolisation, myomectomy and hysterectomy.

**Design:**

Cost-utility analysis based on a Markov model.

**Setting:**

National Health Service (NHS) Trusts in England and Wales.

**Population:**

Women for whom surgical treatment for uterine fibroids is being considered.

**Methods:**

The parameters of the Markov model of the treatment of uterine fibroids are drawn from a series of clinical studies of MRgFUS, and from the clinical effectiveness literature. Health-related quality of life is measured using the 6D. Costs are estimated from the perspective of the NHS. The impact of uncertainty is examined using deterministic and probabilistic sensitivity analysis.

**Main outcome measures:**

Incremental cost-effectiveness measured by cost per quality-adjusted life-year (QALY) gained.

**Results:**

The base-case results imply a cost saving and a small QALY gain per woman as a result of an MRgFUS treatment strategy. The cost per QALY gained is sensitive to cost of MRgFUS relative to other treatments, the age of the woman and the nonperfused volume relative to the total fibroids volume.

**Conclusions:**

A treatment strategy for symptomatic uterine fibroids starting with MRgFUS is likely to be cost-effective.

*Please cite this paper as:* Zowall H, Cairns J, Brewer C, Lamping D, Gedroyc W, Regan L. Cost-effectiveness of magnetic resonance-guided focused ultrasound surgery for treatment of uterine fibroids. BJOG 2008;115:653–662.

## Introduction

Uterine fibroids (leiomyomas or myomas) are benign clonal tumours of the smooth muscle cells of the uterus.^1^,^2^ They are the most common benign tumours in women during their reproductive years, with an estimated cumulative incidence of about 40% for white women aged 35–39, rising to over 60% in women aged 45–49.^3^,^4^ Symptoms attributable to fibroids can be classified into three categories: abnormal uterine bleeding, pelvic pressure and pain, and reproductive dysfunction. Despite the high prevalence, there is considerable debate and uncertainty about the optimum management of uterine fibroids. The wide range of treatment options and the lack of information about natural history, long-term effectiveness, outcomes and costs can make decision-making difficult for the women and clinicians.

Studies of outcomes following hysterectomy indicate high patient satisfaction, improved health-related quality of life and complete resolution of menstrual disturbance without the possibility of recurrence. However, hysterectomy is a major operation that causes considerable disability within 2 months of surgery, has mortality rates in the range of 0.38–1 per 1000, severe complications in 3% of women and minor morbidity in up to 30% women.^3^ Hysterectomy is also thought to be associated with long-term consequences such as urinary incontinence years after the operation, which may cause early ovarian failure, and has significant cost implications. This has stimulated the search for clinically and cost-effective alternatives to hysterectomy, which provide comparable quality of life and fewer adverse effects and complications than hysterectomy.^3^ Established treatments for uterine fibroids include hysterectomy, myomectomy and uterine artery embolisation (UAE); endometrial ablative techniques are among emerging new technologies.

MRgFUS uses a noninvasive thermal ablation device integrated with an MR imaging system for the ablation of soft tissue. Recent applications have included the treatment of breast, liver, brain and metastatic bone cancers.^5^–^10^ The physician acquires a set of MR images, identifies a target volume of tissue to be treated and draws the treatment contours. Therapy planning software calculates the type and number of sonications required to treat the defined region while minimizing total treatment time. During the treatment, a small bean-shaped volume of focused ultrasound energy is directed into the target for approximately 15 seconds and heats the tissue to between 60 and 90°C to induce thermal coagulation. MR images taken during sonication provide a diagnostic quality image of the target tissue and a quantitative, real-time temperature map overlay to confirm the therapeutic effect of the treatment. Typically, 20–50 individual sonications are delivered over a 2-hour period to complete a treatment.

MRgFUS for uterine fibroids (ExAblate 2000; InSightec, Haifa, Israel) was approved by the FDA in 2004 (FDA Report, unpublished). The advantages of MRgFUS over existing thermoablative techniques are that it provides continuous MR imaging of fibroids and adjacent structures such as bowel, bladder and sacral nerves and provides continuous temperature monitoring to optimize effective tissue coagulation to prevent injury to adjacent normal tissue.^5^ The advantages of MRgFUS over UAE are reduced infection rates and febrile morbidity, as the thermal coagulated fibroid tissue is easily absorbed by the body. Overall, MRgFUS is associated with a low risk of postprocedural complications.

## Methods

The aim of this study is to estimate the cost-effectiveness of a treatment strategy for symptomatic uterine fibroids with MRgFUS as compared with current practice comprising UAE, myomectomy and hysterectomy. Results are expressed as incremental cost-effectiveness ratios (ICERs), specifically the cost per quality-adjusted life-year (QALY) gained. All costs and QALYs are discounted at an annual rate of 3.5%.^11^

At present, decision-making regarding fibroids can be difficult primarily due to a paucity of data regarding natural history, effectiveness of available treatments and associated costs. Decision modelling provides a concise and explicit framework to quantify the costs and clinical benefits given the existing data uncertainty. A Markov model is used to simulate the natural history, to provide projections of possible outcomes and to identify areas where additional information is needed to permit informed decisions on the part of providers, payers and patients. It facilitates inclusion of important events, such as progression to further treatment and to menopause, occurring at different times for different women.

**Figure 1:**
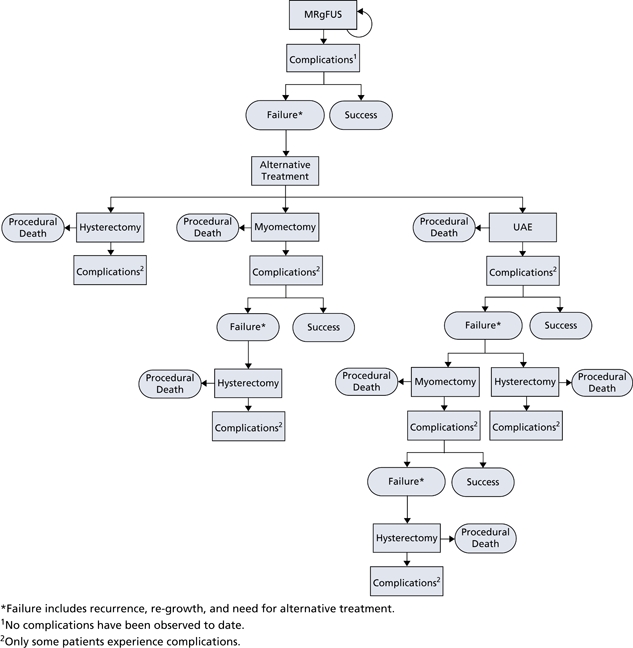
Structure of the model.

Women are assigned discrete health states simulating the clinical outcomes, with corresponding costs and quality of life, and move from one health state to another over time according to preselected transition probabilities. The structure of the model where treatment starts with MRgFUS is presented in Figure 1. Current practice is represented by the same model with MRgFUS omitted. Following an initial fibroid treatment, women can recover from the procedure, with or without short and long-term complications, or die due to the procedure. Those who recover, with or without complications, and require further treatment are classified as failures. Women who do not require further treatment to alleviate fibroid-related symptoms are classified as successes. Treatments can be ordered in terms of increasing invasiveness (MRgFUS, UAE, myomectomy, hysterectomy). Women who fail with their initial treatment proceed over time to a more invasive procedure.

In the base case, the model starts at age 39 and follows women until age 56. There are assumed to be no clinical or cost differences between treatments after menopause. In the absence of UK data on the proportion of women receiving UAE, myomectomy or hysterectomy as their initial treatment for uterine fibroids, it is assumed in the base case that women are distributed across the three treatments, 25% to UAE, 25% to myomectomy and 50% to hysterectomy. All outcomes, except quality of life, are tracked in cycles: for the initial procedures over 6 and 12 months, and yearly thereafter. Quality-of-life estimates are calculated monthly within the first year following procedure and annually thereafter. Women are followed individually for all possible events and only one event can occur per cycle. At the end of each cycle, the woman may either become menopausal or die of other causes unrelated to fibroids. Age-specific death rates from causes other than procedural death are taken from UK Life Tables.^12^

Transition probabilities following MRgFUS were estimated by modelling the relationship between nonperfused volume (NPV) relative to the total fibroids volume and the rate of alternative treatment. NPV is the volume of tissue that does not enhance on contrast MRI following administration of a MR contrast agent. The NPV ratio is a surrogate measure of treatment success. Higher NPV ratios are associated with lower probabilities of recurrence due to fibroid regrowth and a reduced need for additional procedures. Data from InSightec clinical studies (clinical studies included: UF002 (109 participants); UF005 (160 participants); and UF014 (73 participants)) were pooled and separate logistic regressions were estimated for 342 women followed over the first 6 months, 248 women followed over the 6–12-month period with no previous alternative treatment and 143 women followed over the 12–24-month period with no previous alternative treatment. Comparison of the observed and the predicted rates of alternative treatment for different NPV ratios indicates that there is no systematic over- or underprediction by these models.

Progression rates beyond the available data were modelled assuming a constant rate pegged at the 24-month data for a predetermined number of years. Some studies suggest that recurrence occurs only in the first 4 years after initial treatment and that women do not seek additional treatments thereafter.^13^ The transitional probabilities of recurrence from UAE and myomectomy to additional procedures over time were obtained from the literature (references in Table 1).

**Table 1:** Parameter values assumed in base case

	Hysterectomy	Myomectomy	UAE	MRgFUS
Procedure-related death %	0.038^13^	0*	0.02^1^,^3^	0^a^,^c^,^d^
Major complications at 1 year				
%	6.2^14^	6.2*	3.92^15^	0*
Cost	£2,282^19^–^22^	£2,282*	£2,282*	
Minor complications at 1 year				
%	27.1^14^	27.1*	20.6^15^	0^a^,^b^,^d^
Cost	£293^19^–^22^	£293*	£293*	
Long-term complications	6.2^14^	6.2*	3.92^15^	
Duration	2 yrs*	1 year*	1 year*	
Cost	£618^19^–^22^	£0*	£0*	
Cost of outpatient monitoring				
Year 1	£152^19^–^22^	£152*	£398^19^	£398^19^
Subsequent years	£0*	£0*	£0*	£0*
Hospital cost	£2727^19^	£2727*	£2727*	£2000^f^
Annual probability of subsequent hysterectomy		0.0334^17^	0.0303^18^	
Annual probability of subsequent myomectomy			0.0097^18^	
Utility post-treatment	0.757^16^	0.757*	0.783*	0.783^a^
Utility fully recovered or post-menopausal	0.802*	0.802*	0.802*	0.802^a^
% Repeat MRgFUS within 2 weeks				3.88^a^,^b^,^c^,^e^

	0–6 months	6–12 months	12–24 months	After 24 months
% Further treatment (NPV: 60%)	0.80^a^,^b^,^c^,^e^	6.49^a^,^b^,^c^,^e^	3.63^a^,^b^,^c^,^e^	3.63*
% UAE	0.20^a^,^b^,^c^,^e^	2.16^a^,^b^,^c^,^e^	1.82^a^,^b^,^c^,^e^	1.82*
% Myomectomy	0^a^,^b^,^c^,^e^	2.16^a^,^b^,^c^,^e^	0^a^,^b^,^c^,^e^	0*
% Hysterectomy	0.60^a^,^b^,^c^,^e^	2.16^a^,^b^,^c^,^e^	1.82^a^,^b^,^c^,^e^	1.82*

Key: numeral (see references);

* Assumption;

a UF002;

b UF003;

c UF005;

d UF008;

e UF014;

f St Mary's NHS Trust (adjusted for MFF).

An NPV ratio of 60% has been assumed in the base case reflecting current commercial practice (Insightec internal data). Based on the logistic regression analysis, for a NPV ratio of 60%, the recurrence rate following MRgFUS is 0.80% for the 0–6-month follow-up period, 6.49% for the 6–12-month follow-up period and 3.63% for the 12–24-month follow-up period. This compares to a cumulative 5-year recurrence rate of 62% following myomectomy.^23^ The percentage distribution of alternative treatments following MRgFUS among UAE, myomectomy and hysterectomy was taken from the UF005-B2 database. According to current commercial protocols, if needed, the MRgFUS treatment can include a second MRgFUS session within 2 weeks of the initial treatment. Based on the NPV database, 3.88% of the women from the clinical studies have undergone a second MRgFUS session.

Data on short- and long-term complications were obtained from the UF002^5^,^6^ and UF005 studies.^24^ To date, over 3000 women worldwide have been treated with MRgFUS. Safety data from these studies consistently show that there are no FDA reportable adverse events related to MRgFUS. The only device-related adverse events reported include skin burns and nerve damage following MRgFUS, which resolved within a year.^25^ There were no emergency surgical interventions required after MRgFUS. No unexpected short-term adverse events and no long-term complications have been observed to date. Data on outcomes, including procedural death, short- and long-term complications, recurrence rate of alternative treatments and quality of life were taken from the literature (Table 1).

Health-related quality of life following successful treatment is assumed to be the same for MRgFUS and other treatments. This assumption is consistent with the results of recent studies comparing quality of life following hysterectomy versus medical treatment.^26^–^28^ A health state utility of 0.802 was observed at 6 months in the UF002 study. This was derived by converting Short Form(SF)-36 data to the SF-6D.^29^ Quality of life is assumed not to change beyond 6 months post-treatment (based on the absence of any statistically significant improvements in quality of life at 12 and 24 months (in UF008).^7^ This is consistent with Sculpher *et al.* who reported little change in health-related quality of life between 4 months and 1 year after hysterectomy.^30^ Utility following treatment with MRgFUS is 0.783 (based on UF002). Similar utility is assumed following UAE. Utility following hysterectomy (and myomectomy) is assumed to be 0.757 applying the change in utility observed in Garside *et al.*^17^ and assuming that utility at 6 months following successful treatment is 0.802. Quality of life among failures is assumed not to change. Reductions in quality of life due to complications have been estimated from the literature.^15^–^17^

This study assumes an National Health Service (NHS) perspective. Costs to the NHS include initial and subsequent hospitalisations and outpatient services such as day procedures, diagnostic tests, medical personnel, medication costs etc. Since all symptomatic women routinely undergo pre-treatment evaluations before being offered any treatment, these costs are not included in the comparison between treatment strategies.

Hospital costs of MRgFUS are based on estimates of resource use obtained from St Mary's NHS Trust, London including all personnel costs, medical consumables, MR time, equipment and maintenance costs. Because some of the costs are independent of the number of women treated, the cost per woman is sensitive to assumptions regarding the number of women treated. In the base case it is assumed that 220 women are treated annually (on average five women per week for 44 weeks in each year). At this level of patient throughput the estimated cost is £2382 per woman. Such costs are, however, unrepresentative of what the cost of MRgFUS would be elsewhere in the UK. The Department of Health recognizes that providers operating in areas such as London and the South East face higher costs for staff, land and buildings due to external market forces.^20^ To compensate for this, the Market Forces Factor (MFF) adjustment is employed and the MFF for St Mary's NHS Trust is the highest in the country. Accordingly, all costs (other than consumables and equipment costs) have been multiplied by 0.78 (the ratio of the mean MFF for all English NHS Trusts to the MFF for St Mary's). The estimated cost of MRgFUS of £2000 is more comparable to the National Reference Costs for other procedures.

The mean cost of elective inpatient hysterectomy is £2727, with an interquartile range of £2054 and £3157.^20^ Since the literature indicates that there is little variability in the initial hospital costs between UAE, myomectomy and hysterectomy^31^,^32^ the cost of all three is assumed to be £2727. Outpatient medical costs were derived using the 2005 NHS Reference Costs and the 2004 Personal Social Services Research Unit.^20^,^21^ Outpatient medication costs were obtained from the British National Formulary and the Scottish Prescription Cost Analysis.^22^,^33^

The effect of uncertainty in the parameters of the model (such as the definition of current practice, the NPV ratio, age of the woman, procedural deaths, recurrence rates, complications rates, quality of life and treatment costs) were studied using extensive one-way and two-way sensitivity analyses.

Probabilistic simulations were carried out to account for the effect of uncertainty regarding the model inputs on cost-effectiveness. To do this, 20 000 simulations were generated, simultaneously varying the following parameters for all four procedures: recurrence rates, complications rates, procedural death, quality of life and hospital costs. Values for the transitional probabilities and health state utilities were sampled from a beta distribution. The distribution of costs for hysterectomy, myomectomy and UAE was assumed to be log-normal with 0.005 and 99.995% quantiles of £1933 and £3818 (140% of its assumed mean of £2727). The log-normal distribution for cost of MRgFUS was chosen so that the 99.995% quantile was also 140% of its assumed mean (£2000). The resulting 20 000 ICERs indicate the range of outcomes that might be expected given the inherent uncertainty in the underlying data. These data are then used to generate a cost-effectiveness acceptability curve indicating the proportion of simulations for which a particular intervention has a positive net benefit (i.e. it shows the probability that the MRgFUS strategy is cost-effective for different willingnesses-to-pay for a QALY).

All analyses were performed using Microsoft Office Excel 2003 software.

## Results

The results of the base-case scenario are presented in Table 2. The total discounted direct medical costs of 1000 women treated with MRgFUS at age 39 and followed until menopause or age 56 have been estimated at £3,101,644, compared with the cost of £3,396,913 for 1000 women treated with currently available procedures. Thus, the incremental cost of an MRgFUS treatment strategy compared with current treatment, results in a cost saving of £295,269. Moreover, MRgFUS treatment compared with current practice increased total QALYs by 10.658. In the base case, MRgFUS is dominant, that is, has a lower cost and better outcomes than the existing treatment strategy (although the QALY difference per woman is very small).

**Table 2:** Base case and deterministic sensitivity analysis

	MRgFUS		Current Practice					
Scenario #	Cost (£)	QALY	Cost (£)	QALY	Δ Cost (£)	Δ QALY	ICER	Modified Model Parameters
1	3,101,644	10793.874	3,396,913	10783.216	−295,269	10.658	Dominant	Base case
2	3,101,644	10793.874	3,486,202	10783.962	−384,558	9.912	Dominant	Current practice: 33.3% U, 33.3% M, 33.3% H
3	3,101,644	10793.874	3,611,206	10785.005	−509,563	8.868	Dominant	Current practice: 45% U, 45% M, 10% H
4	3,101,644	10793.874	3,664,780	10785.453	−563,136	8.421	Dominant	Current practice: 50% U, 50% M, 0% H
5	3,101,644	12785.761	3,396,913	12773.136	−295,269	12.624	Dominant	Utility following successful treatment 0.95
6	3,101,644	11853.732	3,396,913	11837.439	−295,269	16.292	Dominant	Utility following HYS 0.78 rising to 0.88 at 1 year
7	3,077,224	10794.263	3,279,248	10785.032	−202,023	9.231	Dominant	Recurrence rate for HYS, UAE & MYO halved
8	3,074,941	10794.140	3,242,757	10785.871	−167,816	8.270	Dominant	Zero long-term complications for all procedures
9	3,101,644	10793.509	3,396,913	10783.216	−295,269	10.293	Dominant	MRgFUS complications equal to UAE
10	3,101,675	10794.366	3,397,053	10786.049	−295,378	8.317	Dominant	Zero procedural deaths with HYS and UAE
11	3,650,212	10793.874	4,320,922	10783.216	−670,710	10.658	Dominant	Hospital costs £2382 for MRgFUS, £3151 for UAE, £3715 for MYO & HYS (Central London)
12	2,968,182	10793.874	2,671,423	10783.216	296,759	10.658	£27,845	Hospital costs £2054 for UAE, MYO & HYS
13	3,755,912	10793.874	3,396,913	10783.216	359,000	10.658	£33,685	Hospital costs £2630 for MRgFUS(3 patients per week)

The results of the deterministic sensitivity analysis are summarized in Table 2, scenarios 2–13. In scenario 2, the assumption regarding current practice in the UK for the management of uterine fibroids is changed to UAE (33.33%), myomectomy (33.33%), and hysterectomy (33.33%), rather than the base-case scenario of 25, 25 and 50%, across the three treatments, respectively. MRgFUS remains the dominant strategy. Scenarios 3 (and 4) explore more extreme assumptions, namely that 10% of women (or none) in the current practice group undergo hysterectomy as their initial treatment (the remaining women being equally divided between UAE and myomectomy). MRgFUS remains the dominant strategy. Indeed the cost savings increase as the proportion undergoing hysterectomy falls since the costs of UAE and myomectomy are higher than for hysterectomy once one allows for the additional treatments required by some women.

In the base case, all successful treatments for uterine fibroids are assumed to result in similar improvements in quality of life after the recovery period; that is, all women after successful recovery reach a health state utility value of 0.802 (based on the results for the successfully treated group in UF002). Although it might be argued that quality of life will be lower for those women who have had their ovaries removed. In scenario 5, it is assumed that all women would reach a health state utility value of 0.95, which is the value for full recovery after hysterectomy, reported by Sculpher *et al.*^34^ and used in the Markov model by Garside *et al.*^35^ The QALYs gained as a result of an MRgFUS strategy increase slightly to 12.624 and MRgFUS remains the dominant strategy. Scenario 6 is based on health state utility values used in Hurskainen *et al.*,^26^,^27^ where utility values for hysterectomy are 0.78 at baseline and 0.88 at 1 year,^27^ remaining unchanged at 5 years.^26^ The QALYs gained as a result of an MRgFUS strategy increase to 16.292 and MRgFUS remains the dominant strategy.

The impact of alternative assumptions regarding the effectiveness of the treatments is explored by varying the assumptions made regarding recurrence rates, NPV ratios, complication rates and procedural death rates. In scenario 7, the annual recurrence rates of all procedures other than MRgFUS were decreased by 50%. MRgFUS remains the dominant strategy. When long-term complications for all procedures are reduced to zero (scenario 8), MRgFUS again remains the dominant strategy. When the complication rate for MRgFUS is set equal to that of UAE (scenario 9), MRgFUS is still the dominant strategy. If procedural death rates for UAE (0.0002) and hysterectomy (0.00038) are set to zero (scenario 10), MRgFUS remains the dominant strategy.

The costs of treatment are varied in scenarios 11–13. In scenario 11 central London (St Mary's NHS Trust) costs are assumed for all procedures and MRgFUS remains the dominant strategy. In scenario 12, the costs of all procedures (other than MRgFUS) are assumed to be equivalent to the lower quartile of hysterectomy costs (£2054).^20^ MRgFUS is no longer cost saving and the resulting ICER is £27,845 per QALY. If the initial hospital costs of MRgFUS are increased from £2000 to £2630 (the estimated cost given three patients per week), the cost saving is eliminated and the resulting ICER is £33,685 per QALY (scenario 13).

Predictably, increases in the cost of current treatments or falls in the cost of MRgFUS, increases in the long-term complication rates or annual recurrence rates for current procedures, all leave MRgFUS dominant (with increased estimated cost savings).

**Figure 2:**
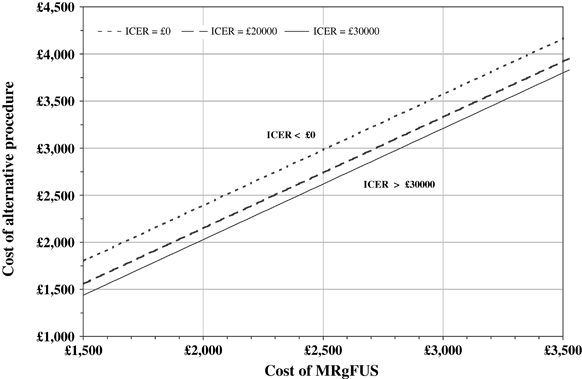
Two-way sensitivity analysis—cost of MRgFUS versus cost of alternative treatment.

A two-way sensitivity analysis varying the cost of MRgFUS and the costs of alternative treatments at the same time is presented in Figure 2. The three lines show the combinations of MRgFUS costs and costs of alternative procedures that would produce ICERs of £0, £20,000 and £30,000. The closeness of these three lines highlights how sensitive results are to assumptions about the relative cost of MRgFUS and of the alternative procedures.

In the base case, women were assumed to be aged 39 years. As the age of women is increased from 35 years to 56 years the cost saving anticipated as a result of an MRgFUS strategy increases, and the QALY gain decreases and then becomes negative. The total QALYs for the successive cohorts fall as starting age rises for both the MRgFUS and current treatment strategies. However, the total QALYs for MRgFUS fall faster than for the current treatment strategy because the predicted QALY stream in the future is higher for MRgFUS than the current strategy (as a result of differences in long-term complications and in procedural mortality). Thus restrictions in the time horizon causes more QALYs to be lost from the MRgFUS strategy compared with the current treatment strategy. Up until age 43, MRgFUS is the dominant strategy. For women aged 44 and above MRgFUS is associated with a loss of QALYs compared with current practice, although MRgFUS remains cost saving. At age 44 the ICER for the current treatment strategy over MRgFUS is £79,863; at age 45 it is £78,677 per QALY. Whereas, at ages 46 and 47 the ICER for the current strategy falls to £23,659 and £21,739. Current treatment is cost-effective for women aged 48 and over.

As the NPV ratio rises, a predicted QALY loss with MRgFUS becomes a predicted QALY gain with no difference in the number of QALYs arising between 25 and 30%. MRgFUS has a positive net cost, which decreases as the NPV ratio increases, and it becomes cost saving between an NPV ratio of 45 and 50%. Thus, up to 25%, MRgFUS is dominated by current practice. Between 30 and 45% MRgFUS produces additional QALYs with a cost per QALY of £304,850 at 30%, and £5040 at 45%. NPV values of 50% and above result in MRgFUS being the dominant strategy with rising positive incremental QALYs and increasing cost savings.

**Figure 3:**
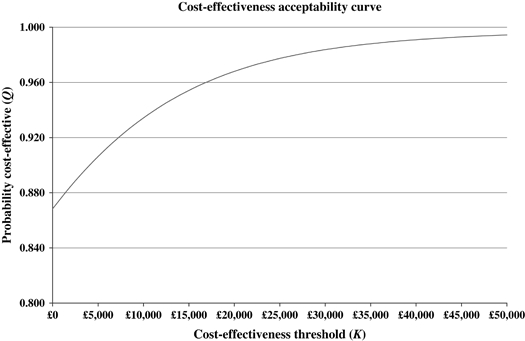
Cost-effectiveness acceptability curve.

Probabilistic simulations were undertaken to account for the effect of uncertainty in the model inputs on cost-effectiveness; 20,000 simulations were performed, simultaneously varying the following parameters for all four procedures: procedural death, recurrence rates, complications rates, quality of life and treatment cost. The results of the probabilistic sensitivity analysis are presented in Figure 3. For approximately 86% of simulations, MRgFUS is dominant (positive incremental QALYs and negative incremental costs). The median solution results in incremental QALYs of 10.173 and cost savings of £285,684.

## Discussion

A treatment strategy for symptomatic uterine fibroids starting with MRgFUS is likely to be cost-effective. In the base case it is dominant, that is, it has a lower cost and better outcomes than the current treatment strategy—on average a cost saving of about £295 and a gain of one hundredth of a QALY per woman. It remains cost-effective under alternative assumptions regarding current practice, health utility estimates before and after treatment and the effectiveness of alternative treatments (complication rates, recurrence rates and procedural death rates).

The cost per QALY gained is sensitive to the cost of MRgFUS relative to other treatments, the age of the woman and the NPV relative to the total fibroids volume. For example, if the lower quartile costs for hysterectomy are assumed or if three women per week (rather than five) undergo MRgFUS, MRgFUS has a cost per QALY gained close to £30,000 rather than being the dominant strategy. MRgFUS is cost-effective up until the age of 45. At ages 46 and 47 the current strategy produces additional QALYs at an incremental cost, which might be viewed as acceptable. However, if a cost-effectiveness threshold of £20,000 per QALY is applied, MRgFUS would be considered the cost-effective treatment strategy.^11^ Finally, the results are sensitive to the NPV ratio achieved by MRgFUS, MRgFUS becomes cost-effective between 40 and 45% and dominant above 50%.

The probabilistic simulations show the range of outcomes that might be expected in practice, given the underlying uncertainty in available data. The finding that MRgFUS is dominant in approximately 86% of simulations provides strong support for its cost-effectiveness. The probabilistic sensitivity analysis confirms the results of the deterministic sensitivity analysis. These results should be treated with appropriate caution recognising, in particular, the limitations of the available cost data.

This is the first study to evaluate the cost-effectiveness of adding MRgFUS technology to the arsenal of existing treatments for uterine fibroids. Data from the available studies of MRgFUS are combined with extensive sensitivity analyses to handle the inevitable uncertainty in the available data. Moreover, all model parameters regarding UAE, myomectomy and hysterectomy are based on recent literature (post-1999).

Despite providing extensive sensitivity analyses, there remains some inherent uncertainty regarding the model's parameters. The model, by necessity, depends on inferred comparisons in the absence of data from RCTs involving head-to-head comparisons of MRgFUS versus current treatments. Such inferred comparisons are subject to bias and confounding and should be viewed with caution. However, in the absence of direct head-to-head comparisons, such analyses are the only suitable approach for assessing cost-effectiveness.

Given the frequency of the tumour and its role in the incidence of hysterectomy, it is remarkable that there have been so few RCTs comparing hysterectomy to other treatment modalities for fibroids.^3^ In a systematic review of 1084 studies on the surgical and nonsurgical management of fibroids in 2002,^36^ the authors were unable to perform meta-analysis because of the lack of consistent data on many important preclinical variables and the use of different outcome measures across studies. In 2006, the Cochrane Library produced a systematic review of the clinical effectiveness of UAE versus hysterectomy and myomectomy based on the results of RCTs.^37^ The review showed a reduction in length of hospital stay and quicker resumption to daily activities with UAE. Patient satisfaction is similar between UAE and surgery (hysterectomy and myomectomy). Similar findings have been reported in a recent trial.^38^ There is the suggestion that UAE is associated with more intraprocedure and postprocedure complications. Thus, UAE results in high rates of failure and the need for additional interventions. The authors concluded that there is a continued need for further research involving larger RCTs and longer follow up.

A US Technology Assessment Evidence Report in 2000^39^ concluded that there is almost no high-quality evidence to reach definitive conclusions regarding the management of uterine fibroids. The fact that there is so little evidence for women, clinicians and policy makers to use in making decisions about the management of such a common condition is striking. They called for future research to improve comparability across studies, to provide data on long-term outcomes and the use and costs of healthcare services in order to establish cost-effectiveness baselines.

This study has not taken account of the reproductive implications of available treatments; the results of the model apply only to women with no desire for future pregnancy. Many women are concerned about preserving their fertility, and decision-making is greatly influenced by this concern; in many cases they may postpone treatment until later in life. However, MRgFUS is believed to be the treatment, which offers a way to preserve fertility.^40^–^42^ If this is the case, there may be additional quality of life gains associated with MRgFUS that were not captured in this study. Thus, inclusion of fertility consequences on quality of life might improve the overall cost-effectiveness of MRgFUS.

Also an NHS perspective has been adopted, had the loss of productivity also been taken into account this would further strengthen the case for a strategy starting with MRgFUS as a result of the cohort experiencing fewer hysterectomies. Valuing time off work using average earnings increases the cost savings from the MRgFUS strategy by more than £500 per woman.

Despite the paucity of data, decisions regarding resource allocation in health care have to be made. A requirement of many regulatory bodies, including those in the UK, is the need to demonstrate the value of a new intervention through a cost-effectiveness analysis. In the context of limited resources and ever-expanding need for healthcare services, determining the value for money of a new intervention is an important consideration for policy makers who have to make choices within a constrained budget. Thus, clinical effectiveness, costs and patient preferences must be weighted when assessing value for money even with imperfect information.^43^,^44^

## Conclusion

The results of this study support the introduction of MRgFUS as a treatment for uterine fibroids. A treatment strategy starting with MRgFUS is potentially more effective and less costly than current practice. However, the degree of uncertainty attaching to this conclusion, primarily reflecting the quality of the underlying data, should be emphasised.

## Disclosure of interests

W.G. and L.R. are Board members of St Mary's Therapy and Imaging Ltd. W.G. is an occasional consultant for InSightec.

## Contribution to authorship

J.C. and H.Z. designed the study, supervised the analysis and interpreted the results. D.L., L.R. and W.G. obtained funding and contributed to the design, analysis and interpretation of results. C.B. performed the statistical analysis and contributed to study design and interpretation. J.C. drafted the article and others commented. All authors have approved the final version.

## Ethics approval

Not sought, since this study is an economic evaluation for which no patient data were collected.

## Funding

This study was supported by an unrestricted grant from InSightec (Haifa, Israel) to the London School of Hygiene and Tropical Medicine (Principal Investigator: D.L.).
